# Direct and indirect effects of environmental factors, spatial constraints, and functional traits on shaping the plant diversity of montane forests

**DOI:** 10.1002/ece3.5931

**Published:** 2019-12-15

**Authors:** Ting Li, Qinli Xiong, Peng Luo, Yubo Zhang, Xiaodong Gu, Bo Lin

**Affiliations:** ^1^ CAS Key Laboratory of Mountain Ecological Restoration and Bioresource Utilization & Ecological Restoration Biodiversity Conservation Key Laboratory of Sichuan Province Chengdu Institute of Biology Chinese Academy of Sciences Chengdu China; ^2^ University of Chinese Academy of Sciences Beijing China; ^3^ State Key Laboratory of Urban and Regional Ecology Research Center for Eco‐Environmental Sciences Chinese Academy of Sciences Beijing China; ^4^ College of Forestry Beijing Forestry University Beijing China; ^5^ The Wildlife Protection Division of the Forestry Department of Sichuan Province Chengdu China; ^6^ Sichuan Forestry and Grassland Inventory and Planning Institute Chengdu China

**Keywords:** abundance, canopy, plant community, richness, shrub, structural equation model, tree

## Abstract

Understanding the relative importance of the factors driving the patterns of biodiversity is a key research topic in community ecology and biogeography. However, the main drivers of plant species diversity in montane forests are still not clear. In addition, most existing studies make no distinction between direct and indirect effects of environmental factors and spatial constraints on plant biodiversity. Using data from 107 montane forest plots in Sichuan Giant Panda habitat, China, we quantified the direct and indirect effects of abiotic environmental factors, spatial constraints, and plant functional traits on plant community diversity. Our results showed significant correlations between abiotic environmental factors and trees (*r* = .10, *p* value = .001), shrubs (*r* = .19, *p* value = .001), or overall plant diversity (*r* = .18, *p* value = .001) in montane forests. Spatial constraints also showed significant correlations with trees and shrubs. However, no significant correlations were found between functional traits and plant community diversity. Moreover, the diversity (richness and abundance) of shrubs, trees, and plant communities was directly affected by precipitation, latitude, and altitude. Mean annual temperature (MAT) had no direct effect on the richness of tree and plant communities. Further, MAT and precipitation indirectly affected plant communities via the tree canopy. The results revealed a stronger direct effect on montane plant diversity than indirect effect, suggesting that single‐species models may be adequate for forecasting the impacts of climate factors in these communities. The shifting of tree canopy coverage might be a potential indicator for trends of plant diversity under climate change.

## Graphical abstract

The correlations of trees, shrubs, or plant composition with the environmental and spatial constraints were significant, while there was no significance for the plant functional traits. There is a stronger direct effect on montane plant diversity than indirect effect. The impact of climate factors on plant diversity might be indirectly affected by global climate change.

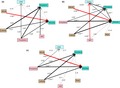

## 1 INTRODUCTION

Understanding the relative importance of the factors driving patterns of biodiversity is an important topic in ecology and biogeography (Gaston, 2009). However, we still do not have a thorough understanding of the factors limiting the patterns of plant community diversity (Heino & Tolonen, 2017; Victorero, Robert, Robinson, Taylor, & Huvenne, 2018), especially in mountain regions over the world (Gaston, 2009).

Plant communities are complex and composed of organisms with very different life history traits, thermal tolerances, and dispersal ability (Classen et al., 2015). Therefore, the diversity of plant communities is not only controlled by external factors (i.e., geography, land cover, and environmental conditions) (Victorero et al., 2018; Xiong et al., 2019), but also by internal factors (biological characteristics, such as life history and functional traits) (Soininen, Lennon, & Hillebrand, 2007). Most studies have generally agreed that variation of environmental factors (including climate change) dominates deterministic processes of plant community composition changes (Heino, Mykrä, Kotanen, & Muotka, 2010; Mykrä, Heino, & Muotka, 2010; Victorero et al., 2018). However, some studies have pointed out that there are significant correlations between species characteristics/traits (i.e., biomass, canopy height, and leaf area) and plant community diversity (Jenni, Janne, & Helmut, 2010; Soininen et al., 2007). Moreover, the effect of environmental changes on species diversity is mediated by plant functional traits (Heino & Tolonen, 2017; Mcgill, Enquist, Weiher, & Westoby, 2006; Verberk, Noordwijk, & Hildrew, 2013). Thus, conducting a study on functional traits may contribute to understanding how environmental conditions filter species from regional species pools and how species compete for resources.

Environmental factors, biological factors, and spatial constraints are interrelated or change together under natural conditions (Mcgill et al., 2006; Verberk et al., 2013). These multiple drivers can interact in ways not predictable by single factor effects to directly influence vital rates of plant diversity through nonadditive effects on demography, physiology, and morphology (Farrer, Ashton, Knape, & Suding, 2014). Indirect effects can be driven either by changes in the abundance of other species or by changes in the direction and/or strength of per capita interaction effects via functional traits (Gilman, Urban, Tewksbury, Gilchrist, & Holt, 2010; Tylianakis, Didham, Bascompte, & Wardle, 2008). For example, climate change may lead to an increase in abundance of one plant species by reducing the abundance of another (reducing competition) (Li et al., 2020). In addition, these biotic interactions may covary with environmental gradients, further confounding our understanding of the true strength of the abiotic and biotic drivers (Chu et al., 2019). Given the potential importance of indirect effects, ignoring biotic interactions could severely affect the accuracy of forecasts of species abundances and distributions under a changing environment (Angert, LaDeau, & Ostfeld, 2013; Chu et al., 2019), consequently limiting the effectiveness of conservation and management actions. However, few studies have addressed the importance of direct effects (single factor or interactive) and indirect effects of driving factors on the species diversity and distribution of plant communities. Indirect effects of environmental variables on plant community can be strong in High Arctic or alpine ecosystems (Dormann, Wal, & Woodin, 2004; Klanderud & Totland, 2005), whereas direct effects can predominate in other ecosystems (Levine, McEachern, & Cowan, 2010). Alternatively, mechanisms might be largely site‐specific, varying in both direct and indirect drivers owing to unique climatic conditions and management history.

Vegetation composition and patterns in montane forests may be influenced by direct and/or indirect drivers of different environmental factors, geography, and biological characteristics (Chu et al., 2019; Li et al., 2020). Montane forests are characterized by high topographic variability in a small area; this variability includes topographical factors such as elevation, slope inclination, and ground surface texture (Andrus, Harvey, Rodman, Hart, & Veblen, 2018). In addition, the vegetation in montane forests is highly sensitive to climate change with multiple functional traits (Dakhil et al., 2019; Xiong et al., 2016). Because of these abiotic and biotic differences, plant diversity might vary in terms of their responses to multiple drivers, especially when studied as a network of indirect and direct drivers of diversity. With field data from a multisite survey, we identify direct and indirect pathways linking multiple drivers to plant diversity in montane forests. We hypothesize that (a) spatial and climate factors, such as mean annual temperature (MAT), precipitation, and spatial distance, are the main drivers of plant species diversity; (b) functional traits play a small but significant role in plant diversity; and (c) overall responses to spatial and climate drivers are driven primarily by direct effects. The aims of this research were to (a) identify the main factors influencing the interrelationships between environmental, spatial, and functional traits, and explore the causes that lead to a change in plant diversity and (b) disentangle the direct and indirect effects of environmental, spatial, and functional traits on species diversity within a plant community in montane forests. By characterizing the complexity of diversity responses to multiple drivers, we aim to contribute to a predictive understanding of how direct and indirect effects drive the variability of plant diversity in montane forests.

## 2 MATERIALS AND METHODS

### 2.1 Study area

This study was conducted in the Sichuan Giant Panda habitat, which is located in an alpine valley in the transition region from the Qinghai–Tibetan Plateau to the Sichuan basin (Li et al., 2019). The area is part of the subtropical evergreen broadleaf forest region and warm temperate deciduous broadleaf forest region (Sichuan Vegetation Cooperation Group, 1980). The Sichuan Giant Panda habitat was established as a UNESCO World Heritage Site in 2006. It is a refuge to diverse wildlife and plant species, as well as home to more than 30% of the wild giant panda population (State Forestry Administration, 2006). In fact, the region is within one of the world's top 34 biodiversity hotspots (Bellard et al., 2014) and one of the Global 200 Ecoregions defined by the World Wildlife Fund (WWF) (Dakhil et al., 2019).

### 2.2 Plant survey

In 2017, 107 random sampling plots in montane forests were collected from north to south, spanning the entire Sichuan Giant Panda habitat. The sampling strategy and field site information are shown in Li et al. (2019). The elevation within the sampling plots varied significantly (from ca. 2,000 to 3,600 m a.s.l.) (Li et al., 2019). The main vegetation types in those plots were coniferous and broad‐leaved mixed forests, and evergreen and deciduous broad‐leaved mixed forests. Using questionnaires, we surveyed 72 local people from Minshan, Xiaoxiangling, and Qionglai in the Sichuan Giant Panda habitat in 2017. Those local villagers mainly participated in the local Giant Panda habitat conservation. The survey information included if there was any interference in the sampling plots. In addition, we observed the plant species composition and environment in the montane forests to choose only mature forests. We finally screened 107 mature forest sampling plots without human interventions. Vegetation surveys were conducted between July and September 2017 (the peak period of plant growth). All plots were located at least 150 m from the road to avoid edge effects. Within each plot, trees in a 20 m × 30 m subplot and shrubs from three 5 m × 5 m subplots were studied. Data from the three subplots within each plot were then pooled. The plant species, number of individuals (abundance), and coverage of each layer (e.g., tree, shrub) were recorded (Table S1).

### 2.3 Variables of environmental, spatial constraints, and plant functional traits

A total of five environment factors were recorded for each sample (Li et al., 2019): altitude, slope, aspect, mean annual temperature (MAT), and precipitation. Meteorological data were mainly obtained from in situ monitoring in each protected area.

Variables of spatial constraints mainly include longitude, latitude, and spatial distance (Li et al., 2019). The spatial variables that constrain the ordinate model were provided based on Moran's eigenvector (MEM) (Dray, Legendre, & Peres‐Neto, 2006). In order to avoid spatial autocorrelation, we used Moran's I index to extract 90 spatial variables with positive eigenvalues and negative correlations. The MEM spatial variable was obtained by the Principal Coordinates of Neighbor Matrices (PCNM) function in the PCNM package. The spatial distance between the sample sites was calculated by the longitude and latitude in the geosphere package in R (Legendre, Borcard, & Peres‐Neto, 2012).

Seven plant functional traits, namely tree canopy coverage, diameter at breast height (DBH), canopy height, specific leaf area, leaf area (LA), leaf thickness (LT), and leaf dry matter content (LDMC), were measured at each study site (Li et al., 2019). These traits reflect the substance exchange balance between plant resource acquisition and protection under environmental change (Bernard‐Verdier et al., 2012). We screened mature plant individuals without pests and diseases. For each species, we randomly selected five individuals and repeated sampling five times for each selected individual (Pérez‐Harguindeguy et al., 2013). The functional trait data were obtained from those samples in the plot. The functional traits of the main dominant tree and shrub species were presented as a weighted average, which was used to investigate the functional traits of the plant community as a whole.

### 2.4 Statistical analysis

A Pearson correlation analysis was conducted on plant alpha diversity, environmental factors, functional traits, and spatial constraint variables. High collinearity factors from these variables were excluded (Li et al., 2019). A principle component analysis (PCA) was applied on the selected variables for spatial, environmental, and functional traits. A Mantel correlation test was used to determine the Sorensen matrix of the composition of trees, shrubs, and overall plant species with an impact factor matrix (Euclidean distance) composed of environmental variables, spatial variables, and plant functional characteristics.

Structural equation models were employed to analyze the environment variables, spatial constraint variables, and functional trait variables (a total of 15 variables, Li et al., 2019) for richness and abundance. To develop the final SEMs, we started with our initial hypothesized relationships among the variables. The decision to remove a path was based on the performance of overall model fit and the *p*‐value for the path (Grace, 2006). To simplify the SEMs, we first deleted the influencing factors with collinearity according to the results of the previous correlation analysis (Li et al., 2019). We did not establish the relationship between climatic factors and topographic factors (elevation, slope, and slope direction), because climatic factors had limiting affect on topography in decades in this area (Xiong et al., 2019). In addition, we hypothesized that climatic variables would significantly affect plant functional traits and preserve the inclusion of functional traits (*p* < .05) in the optimal model. If climatic factors did not significantly affect plant functional traits or if their addition led to a decrease in the best model interpretation, we deleted the correlation between climatic factors and plant functional traits. We used the “lavaan” package in R to model the structural equation (Rosseel, 2012).

Model evaluation was determined by the chi‐square (*χ* ^2^) test (*p* > .05 for a satisfactory fit) and the standardized root mean square residual (SRMR < 0.05 for a satisfactory fit). The Akaike information criterion (AIC) was used to select the best model with a satisfactory fit. When a model met the criteria of the chi‐square test and SRMR but contained nonsignificant paths, we repeated the modeling fit and evaluation by removing these paths. Therefore, the final selected model may not have a minimum AIC value (Li et al., 2019). The decision to remove a path was primarily based on the *p*‐value for the path and the performance of the overall fit of the model. The total effect that one variable had on another equaled the sum of its direct and indirect effects through directed (causal) paths. The *SE* values and *p*‐values for standardized path coefficients were obtained through the function standardized solution in the “lavaan” package of R.

To further understand the response of plant alpha diversity to selected functional traits, climate change, and altitude from SEMs, we analyzed the changes in richness and abundance of shrubs, trees, and the overall plant community through a general linear model (GLM) with a Poisson family distribution using the selected functional traits, climate change, and altitude factor as the dependent variable and species richness/abundance as the response variable.

## 3 RESULTS

### 3.1 Environmental and spatial constraints affecting plant community composition

**Figure 1:**
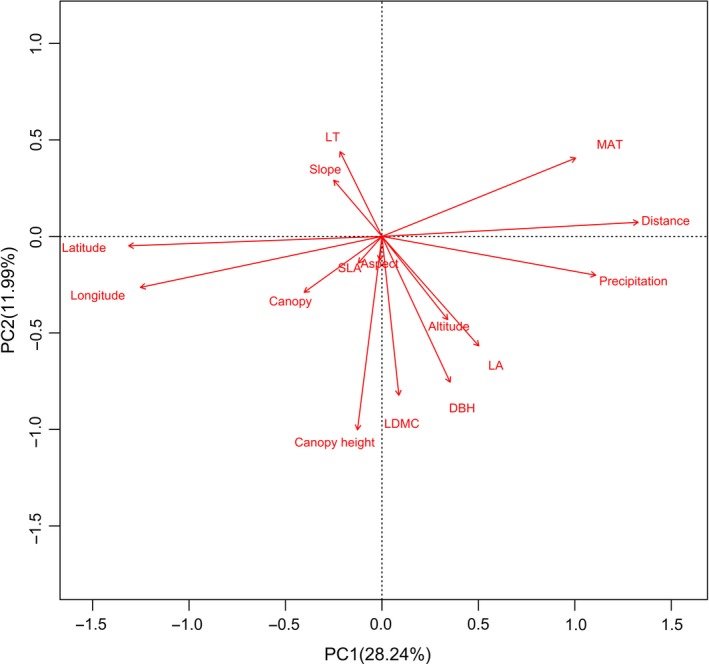
Principle component analysis (PCA) of the selected spatial, environmental, and functional trait variables

The first two axes (PC1 and PC2) of PCA (Figure 1) explained 28.24% and 11.99% of the 15 explanatory variables (Li et al., 2019), respectively. The first axis was mainly composed of environmental factors and spatial constraint variables, including latitude, longitude, spatial distance, precipitation, and MAT (Figure 1). The second axis primarily contained the functional trait and topographic variables, including tree coverage, canopy height, DBH, LA, LT, LDMC, altitude, and slope (Figure 1).

The Mantel correlation analysis showed that the dissimilarity matrix of tree species diversity was significantly correlated with total impact factor (environmental factors, spatial constraints, and functional trait) matrices (*r* = .13, *p* value = .001) (Li et al., 2019). There was a significant correlation between tree species diversity and environmental factors (*r* = .10, *p* value = .001) and a significant correlation between tree species diversity and spatial constraints (*r* = .32, *p* value = .001). Moreover, the species diversity of shrubs was significantly correlated with the total impact factor matrix (*r* = .20, *p* value = .001) (Li et al., 2019), spatial matrices (*r* = .22, *p* value = .001), and environmental matrices (*r* = .19, *p* value = .001). In addition, there was a significant correlation between total plant species diversity and total impact factor matrices (*r* = .23, *p* value = .001). There was a significant correlation between plant community species matrix, spatial matrix (*r* = .36, *p* value = .001), and environmental matrices (*r* = .18, *p* value = .001) (Li et al., 2019). There was no significant correlation of trees, shrubs, or overall plant diversity with the plant functional trait matrix.

### 3.2 Direct and indirect effects of spatial, environmental, and functional traits on plant diversity

**Figure 2:**
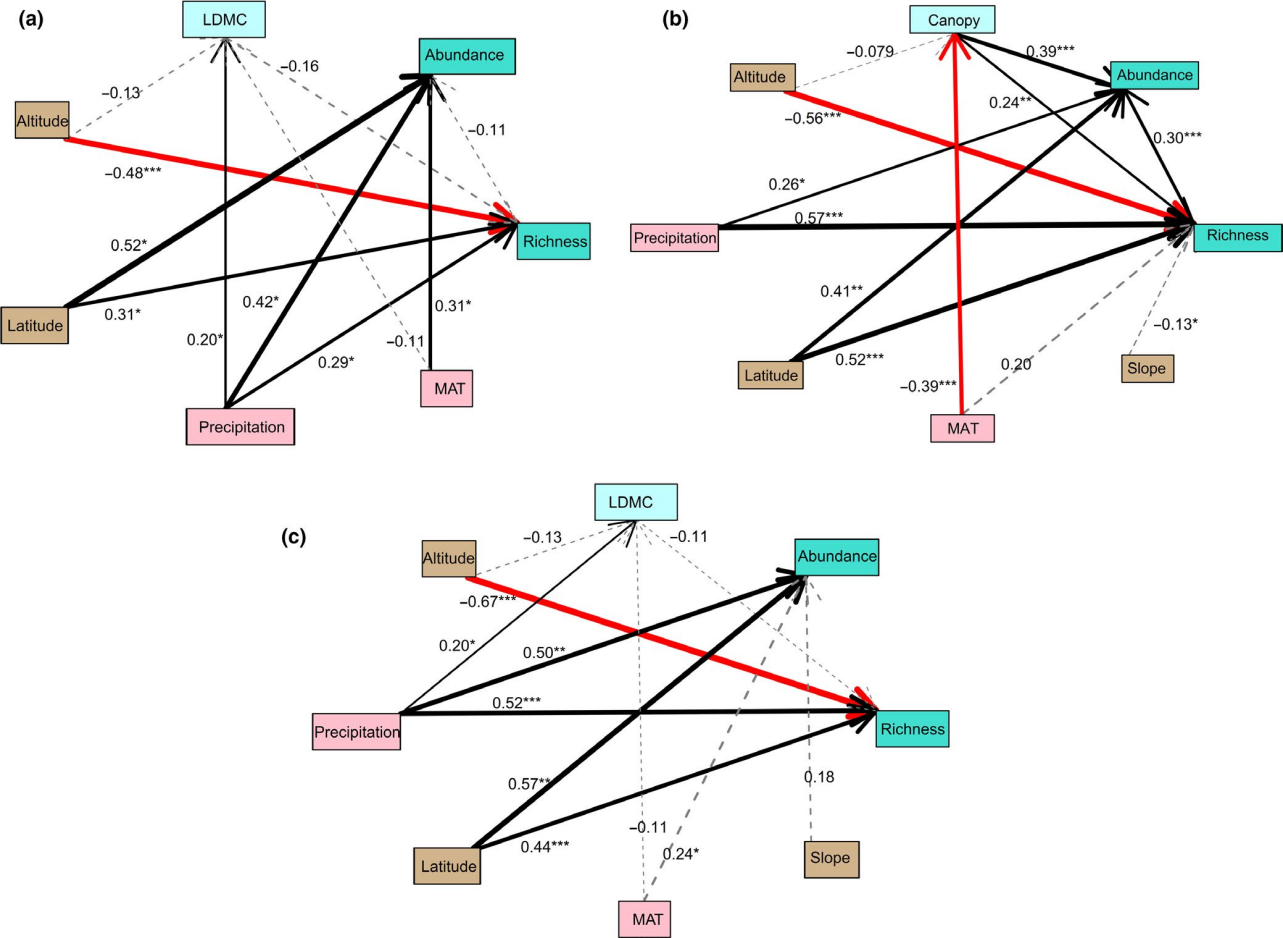
Fitted multigroup structural equation model (SEM) depicting the effects of environmental and biological variables on species richness. Single‐headed arrows represent causal relationships. Numbers on arrows and width of arrows correspond to standardized path strength. A variable lacking a significant relationship with other variables in the model is presented in gray. (a) Shrubs, (b) trees, and (c) plant community

In total, there were 233 shrub species and 174 tree species in the field sites. Because the spatial distance is autocorrelated with the latitude and longitude, we excluded the spatial distance in the SEM analysis. The best SEM (Figure 2) explained the species richness of shrubs as 27.78% variance (*df* = 4, *p* = .41 > .05, SRMR = 0.024 < 0.05), the abundance of shrubs as 6.63%, and the LDMC as 4.80%. Latitude (0.31), altitude (−0.48), and precipitation (0.29) significantly directly impacted the species richness of shrubs, whereas LDMC (−0.16) had no direct impact on shrub richness in the best fit model. Latitude (0.52), precipitation (0.42), and temperature (0.31) significantly directly affected the species abundance of shrubs, while the richness of shrubs (−0.11) had no significant impact on the species abundance of shrubs. LDMC was not directly or significantly affected by temperature (−0.11) and altitude (−0.13), but was significantly affected by precipitation (0.20).

The best SEM explained tree species richness as 52.75% variance (*df* = 6, *p* = .40 > .05, SRMR = 0.036 < 0.05), tree species abundance as 48.58% variance, and tree canopy as 14.37% variance. Latitude (0.52), altitude (−0.56), precipitation (0.57), slope (−0.13), and tree canopy (0.24) significantly directly affected tree species richness, while MAT (0.20) had no significant effect on tree richness in the best fit model. The tree canopy was significantly directly affected by MAT (−0.39), whereas altitude (−0.079) did not significantly affect canopy. Tree species abundance was significantly directly affected by tree richness (0.30), latitude (0.41), precipitation (0.26), and tree canopy (0.39). MAT via tree canopy coverage indirectly affected the abundance ([−0.39]*0.39) and richness ([−0.39]* 0.24) of trees (Tables 1 and 2).

**Table 1:** Direct, indirect, and total effect of precipitation, mean annual temperature (MAT), and latitude on plant species richness in shrubs, trees, and plant communities based on standardized values of statistically significant (*p* < .05) structural equation model (SEM) paths

Dominant effect	Shrubs	Trees	Plant community
Precipitation			
Direct	0.29	0.57	0.52
Indirect	–	–	–
Total	0.29	0.57	0.52
MAT			
Direct	–	–	–
Indirect	–	MAT, Canopy, [−0.39]*0.39	–
Total	–	−0.15	–
Latitude			
Direct	0.31	0.52	0.44
Indirect	–	–	–
Total	0.31	0.52	0.44
Canopy			
Direct	–	0.24	–
Indirect	–	–	–
Total	–	0.24	–

**Table 2:** Direct, indirect, and total effect of precipitation, MAT, and latitude on plant species abundance in shrubs, trees, and plant community based on standardized values of statistically significant (*p* < .05) SEM paths

Dominant effect	Shrubs	Trees	Plant community
Precipitation			
Direct	0.29	0.57	0.52
Indirect	–	Precipitation, richness, 0.57*0.30	–
Total	0.29	0.74	0.52
MAT			
Direct	–	–	–
Indirect	–	MAT, Canopy, [−0.39]*0.39; MAT, canopy, richness [−0.39]*0.24*0.30	–
Total	–	−0.18	–
Latitude			
Direct	0.31	0.52	0.44
Indirect	–	0.52*0.30	–
Total	0.31	0.68	0.44
Canopy			
Direct	–	0.24	–
Indirect	–	–	–
Total	–	0.24	–

The best SEM explained plant community species richness as 50.26% variance (*df* = 6, *p* = .69 (>.05), SRMR = 0.022 (<0.05)), the abundance variance as 10.72%, and LDMC variance as 4.80%. Latitude (0.44), altitude (−0.67), and precipitation (0.52) significantly directly affected plant community richness, whereas LDMC (−0.11) had no significant impact on plant community richness in the best fit model. The species abundance of the plant community was significantly directly affected by latitude (0.57), precipitation (0.50), and MAT (0.24), but slope (0.18) did not significantly affect the plant community abundance in the optimal model. LDMC was not directly affected by MAT (−0.11) and altitude (−0.13); however, precipitation (0.20) significantly directly affected the LDMC.

### 3.3 Effects of functional traits, climate, and latitude gradient on spatial variation of plant diversity

MAT, precipitation, and LDMC did not significantly influence shrub richness, whereas latitude (*Z* value = 2.72, *p* = .0065) significantly affected shrub richness. MAT (*Z* value = 6.23, *p* < .001), precipitation (*Z* value = 3.36, *p* < .001), latitude (*Z* value = 1.90, *p* = .058), and LDMC (*Z* value = −2.51, *p* = .012) significantly affected shrub abundance.

**Figure 3:**
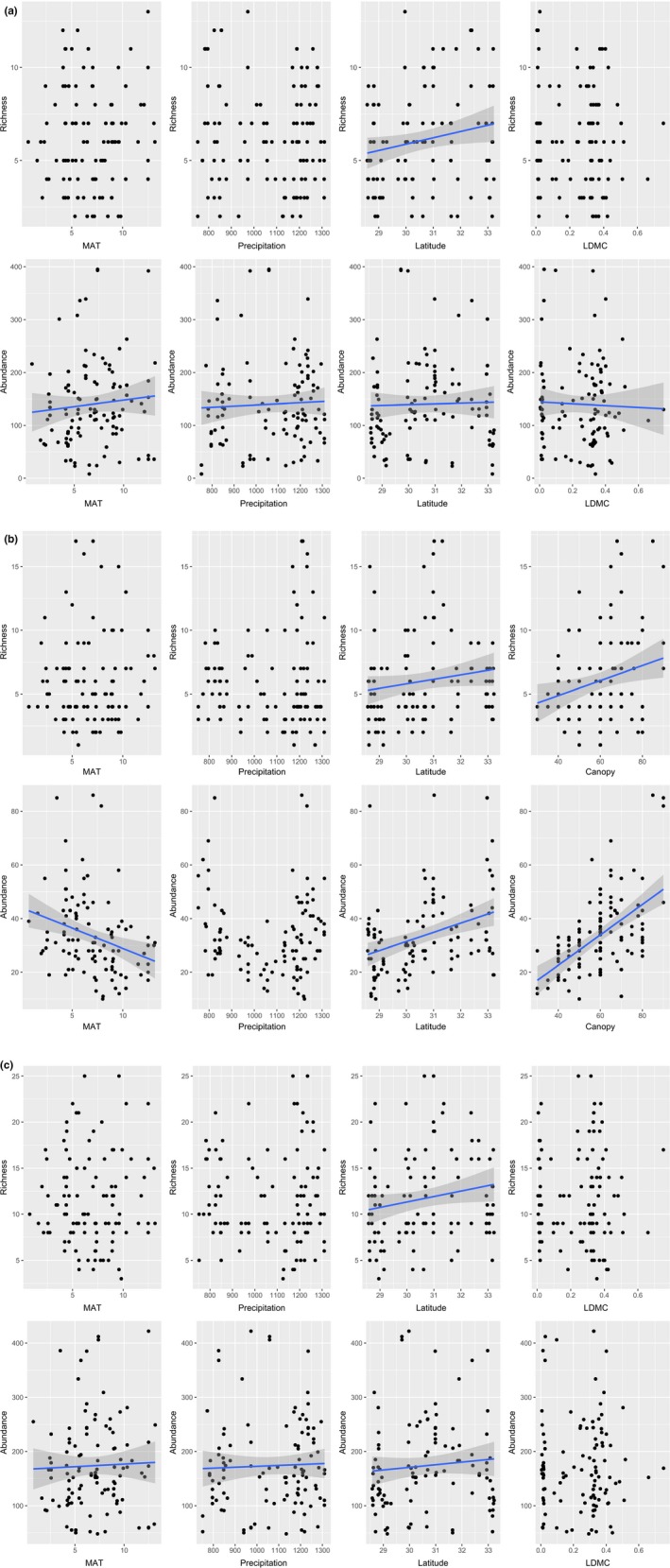
Relationship between plant alpha diversity and selected functional traits, climatic factors, and latitude gradient from SEMs. (a) Shrubs, (b) trees, and (c) plant community

MAT and annual precipitation did not significantly affect tree species richness (Figure 3). Latitude (*Z* value = 2.275, *p* = .023) and the coverage of the tree canopy (*Z* value = 3.54, *p* < .001) significantly affected tree richness. MAT (*Z* value = 7.76, *p* < .001), the coverage of the tree canopy (*Z* value = 14.33, *p* < .001), and latitude (*Z* value = 9.23, *p* < .001) significantly affected tree abundance, while precipitation did not affect tree abundance (Figure 3).

MAT, precipitation, and LDMC did not significantly affect the species richness of the plant community; however, latitude did have a significant effect (*Z* value = 2.16, *p* = .031). MAT (*Z* value = 2.18, *p* = .029), precipitation (*Z* value = 2.32, *p* = .021), and latitude (*Z* value = 5.77, *p* < .001) significantly affected plant community abundance.

## 4 DISCUSSION

In the forest ecosystem of the Sichuan Giant Panda habitat, we found clear evidence that environmental variables and spatial variables both directly and indirectly affected plant species diversity. With a conservative estimate of 233 shrub species and 174 tree species, this study showed significant correlations between abiotic environmental factors, spatial constraints, and plant communities (trees and shrubs), while no significant correlations were found between functional traits and plant communities. Moreover, the diversity (richness and abundance) of shrubs, trees, and plant communities was directly affected by precipitation, latitude, and altitude. However, MAT had no direct effect on the richness of tree and shrub communities. Further, MAT and precipitation indirectly affected species richness via the tree canopy. Our approach provided additional insights into underlying ecological relationships.

### 4.1 Spatial and environmental variables primarily drive the spatial change of plant diversity

Studies support that the pattern of biodiversity of an ecosystem is influenced by a variety of local to regional factors (Heino et al., 2010; Mykrä et al., 2010). The importance of these factors for biodiversity patterns may depend on the spatial dimension of the study area and the characteristics of the species diversity (Heino et al., 2010). Climate is a key driver of the composition of subalpine plant communities (Pauli, Gottfried, Reiter, Klettner, & Grabherr, 2007; Xiong et al., 2016). Our results indicated that MAT and precipitation are important influencing factors that can describe the spatial shifting of plant species diversity; in addition, climate variation can be used as a predictor for the pattern of diversity of montane plant species. Relevant studies have proposed that climate has a strong filtering effect on plant communities (Soininen et al., 2007). In our study, although climate factors had low explanatory power for plant communities (Figure 1), it should be noted that other environmental variables, such as soil moisture, pH, and total nitrogen, have often been found to be critical in determining plant community composition in subalpine montane forests (Hettenbererova, Hajek, Zelený, Jirouskova, & Mikulaskova, 2013). More explanatory variables may be needed to account for the changes in species diversity.

We found that environmental variables and spatial constraints largely explained the diversity of trees, shrubs, and plant communities, while plant functional traits contributed low explanation for community species diversity in montane forests (Figure 1 and Li et al., 2019). Moreover, spatial and environmental variables could explain more trees diversity changes than shrubs. Similar results from a previous study also showed that spatial factors and local environmental variables determined the diversity of stream macroinvertebrates (Perez Rocha et al., 2018). Species diversity may be affected by diffusion dynamics (Leibold et al., 2010). According to previous studies, spatial factors may be related to diffusion restrictions and could play an important role in determining plant species diversity on a broad spatial scale (Mykrä et al., 2010). Therefore, considering the spatial area covered in our study, the decentralized restriction on the regional scale may also drive the change of plant diversity to some extent. In addition, spatial distance and altitude significantly affect plant species diversity, which also indicates that spatial factors limit the ability of plants, especially tree species, to spread over a great distance. In this study, samples were collected in a zonal pattern, showing a significant latitude gradient from the northern edge to the southern edge of the Sichuan Giant Panda habitat. Although the zonal gradient here is not at a global scale, it shows that the spatial change of species diversity is significantly correlated with the climatic and spatial gradient. Our results are consistent with the findings described by Svenning, Fløjgaard, and Baselga (2011), namely, that spatial factors are the most important driving factors for spatial changes in species composition, followed by climatic factors, including current and past climatic factors. Climatic factors are significantly negatively correlated with longitude and latitude, indicating that the plant species diversity in the Sichuan Giant Panda habitat varies significantly along the temperature and precipitation gradient in the north–south direction. Although this may primarily be due to the limited ability of plants to spread, temperature and precipitation conditions are additionally considered to limit the migration ability and adaptability of plants.

Some studies have suggested that biotic interactions may be just as influential in shaping plant community diversity and composition (Warren & Bradford, 2011). However, our findings showed that functional traits may not be as strong drivers of diversity in montane forests as in grasslands or mesic forests (Warren & Bradford, 2011; White, Bork, & Cahill, 2014). The use of plant functional traits may allow for more informative comparisons with regard to gauging ecosystem integrity (Warren & Bradford, 2011). These biotic interactions may covary with environmental gradients, further confounding our understanding of the true strength of the abiotic drivers of plant community diversity and composition. However, previous studies have found it difficult to distinguish between biotic and abiotic drivers (Hettenbererova et al., 2013). Plant functional traits mainly reflect the response of plant species diversity to the change of resource substances. Relevant studies have also proposed that plant height, specific leaf area, and species abundance are strongly correlated, which is consistent with the theory of plant resource acquisition (Heino & Tolonen, 2017). In our study, plant functional traits could not explain the change in plant species diversity; however, tree canopy, the LDMC, and LT were significantly associated with plant diversity, indicating that the abundance of some species changes with plant canopy, LDMC, and leaf thickness. This may be because these factors would allow for the capture of more resource material, proving beneficial for competition. For example, a species with a larger canopy can obtain more light resources, thereby becoming a dominant species in the community and increasing its distribution.

### 4.2 Direct and indirect effects of driving factors on plant community diversity

In our study, SEMs were employed to distinguish direct and indirect effects of environmental factors and biotic interactions on the dynamics of plant diversity. The diversity (richness and abundance) of shrubs, trees, and plant communities was directly affected by precipitation, latitude, and altitude. However, MAT has no direct effect on the richness of trees and plant communities. As previously reported, the effects of temperature on species richness are direct rather than indirect in grasslands (White et al., 2014). Moreover, Hoeppner and Dukes (2012) reported negative responses of richness to warming and provided evidence indicating resistance of grassland diversity to the direct effect of warming. This may be due to the delayed response of perennial trees to temperature changes (Xiong et al., 2016). Moreover, it has been found that trees and plant communities in montane forests are not sensitive to temperature changes over a 10,000‐year time scale (Dakhil et al., 2019), indicating that warming is not the most important factor affecting plants, especially perennials. The tree canopy directly affected the species richness and abundance of trees, while other functional traits had no significant direct impact on plant diversity. MAT and precipitation via the tree canopy indirectly affected the richness and abundance of trees (Figure 2). Warming can indirectly affect diversity through ecological factors such as by altering species interactions or through the plant canopy (Farrer et al., 2014). Our data agree with the numerous studies citing precipitation and latitude as important for the dynamics of plant diversity (Dakhil et al., 2019; Mykrä et al., 2010). Specifically, we found precipitation and latitude consistently important in all three models (Figure 2).

Our results indicate that environmental factors have direct effects related to increasing plant community species richness and abundance. Precipitation has been reported to promote the richness of trees and shrubs, and increasing MAT elevated the richness of shrubs in subalpine mountains as well as grasslands (Chu et al., 2019; Lin, Xia, & Wan, 2010; Xiong et al., 2019). The results from the SEMs showed that MAT may decrease the tree canopy and indirectly inhibit tree diversity (Figure 2). Environment affects the abundance of plant species via tree richness, which was supported by results presented by Storch, Bohdalková, and Okie (2018), but not fully explained by the species energy hypothesis. Here, environmental factors had mainly positive effects on plant community richness. This study found that the direct effects of environmental factors, spatial constraints, and functional traits on the pattern of montane plant diversity were significantly greater than the indirect effects. As observed in our study, precipitation generally had a positive relationship with plant richness (White et al., 2014). Overall responses to climate change are primarily driven by direct effects, suggesting that the response of dominant/single species to climate change may be adequate for forecasting the impacts of climate change within specific communities. The size of the indirect effects also depends on the size of the direct effects experienced by species in the community (Chu et al., 2019). For the differences in the direct and indirect responses of trees and shrubs to environmental factors, ecological niche differences may influence the magnitude of indirect effects. Another factor contributing to variability in the size of raw indirect effects is asymmetry in interspecific interactions (Kleinhesselink & Adler, 2015). The results also illustrate the diversity of species’ responses to environmental variation. However, the relative importance of indirect effects to direct effects could change across the range of a species. Some studies have reported that indirect effects of climate change can amplify, outweigh, or even reverse direct effects (Suttle, Thomsen, & Power, 2007; Tylianakis et al., 2008).

Our findings also indicate some promising future directions. First, the potential importance of indirect effects (the mediating effect of functional traits and biotic interactions) was ignored. Solely focusing on the direct effects of biotic interactions could lead to a severe underestimation of the effect on species abundances and distributions under a changing climate (Angert et al., 2013), consequently limiting the effectiveness of conservation and management activity. Second, the inclusion of additional functional traits, such as foliar profile, representing the vertical dimensions of forest structure, remains a promising area for additional studies. Further, the considerable unexplained variance found in this study suggests that other unmeasured factors (e.g., the abundance of herbivores and pathogens, soil properties) may play a greater role in determining species richness in these forests (Xiong et al., 2016, 2019).

## 5 CONCLUSIONS

By using a framework capable of identifying both direct and indirect responses, we determined the primary drivers of plant richness and abundance in montane forests: climatic factors and spatial constraint (latitude). Our results demonstrate that interactions among environmental factors, spatial constraints, and functional traits both directly and indirectly influence plant species richness in montane forests. The correlations of trees, shrubs, or plant composition with the environmental and spatial constraints were significant, while there was no significance for the plant functional traits. Spatial constraint variables were the main driving force shifting plant species diversity. Moreover, the diversity of shrubs, trees, and plant communities was directly affected by precipitation, latitude, altitude, and the tree canopy. However, MAT had no direct effect on the richness of trees and plant communities. MAT and precipitation via the tree canopy indirectly affected tree richness and abundance. Our results also found that the direct effect was significantly stronger than the indirect effect. Precipitation generally had a positive relationship with plant richness. These findings show that a number of mechanisms act in concert to shape the environmental gradient related to plant diversity, with no single mechanism being sufficient on its own. Our results also illustrated the complexity of ecosystem responses that could unfold following seemingly simple modifications of single factors. However, the factors underlying variability between systems may contribute to predicting how systems will respond, which can be identified by understanding the key drivers of system responses. These findings show that the impact of climate change on plant diversity might be indirectly predicted by the effects of climate change on tree canopy coverage.

## CONFLICT OF INTEREST

The contributing authors declare no conflict of interest regarding the publication of this article.

## AUTHOR CONTRIBUTION

All authors worked together to design this study. T.L. and X.G. collected the data. T.L. and Q.X. carried out the analyses. Q.X., T.L., and P.L. wrote the draft of this paper. All authors contributed considerably to revising and editing this paper.

## Data Availability

Data from this study are archived in the public archive Dryad (http://datadryad.org) at the https://doi.org/10.5061/dryad.4f4qrfj7m (Li et al., 2019).
