# Supplementary material for: Direct and indirect effects of environmental factors, spatial constraints, and functional traits on shaping the plant diversity of montane forests
Source: Ecol Evol. 2019 Dec 15;10(1):557–68. doi: 10.1002/ece3.5931 (PMC6972828; doi:10.1002/ece3.5931)
Supplement: Supplementary file 1 [file ECE3-10-557-s001.docx]

**Fig. S2** Bivariate relationships among all variables for SEMs in the forest plots analyses.


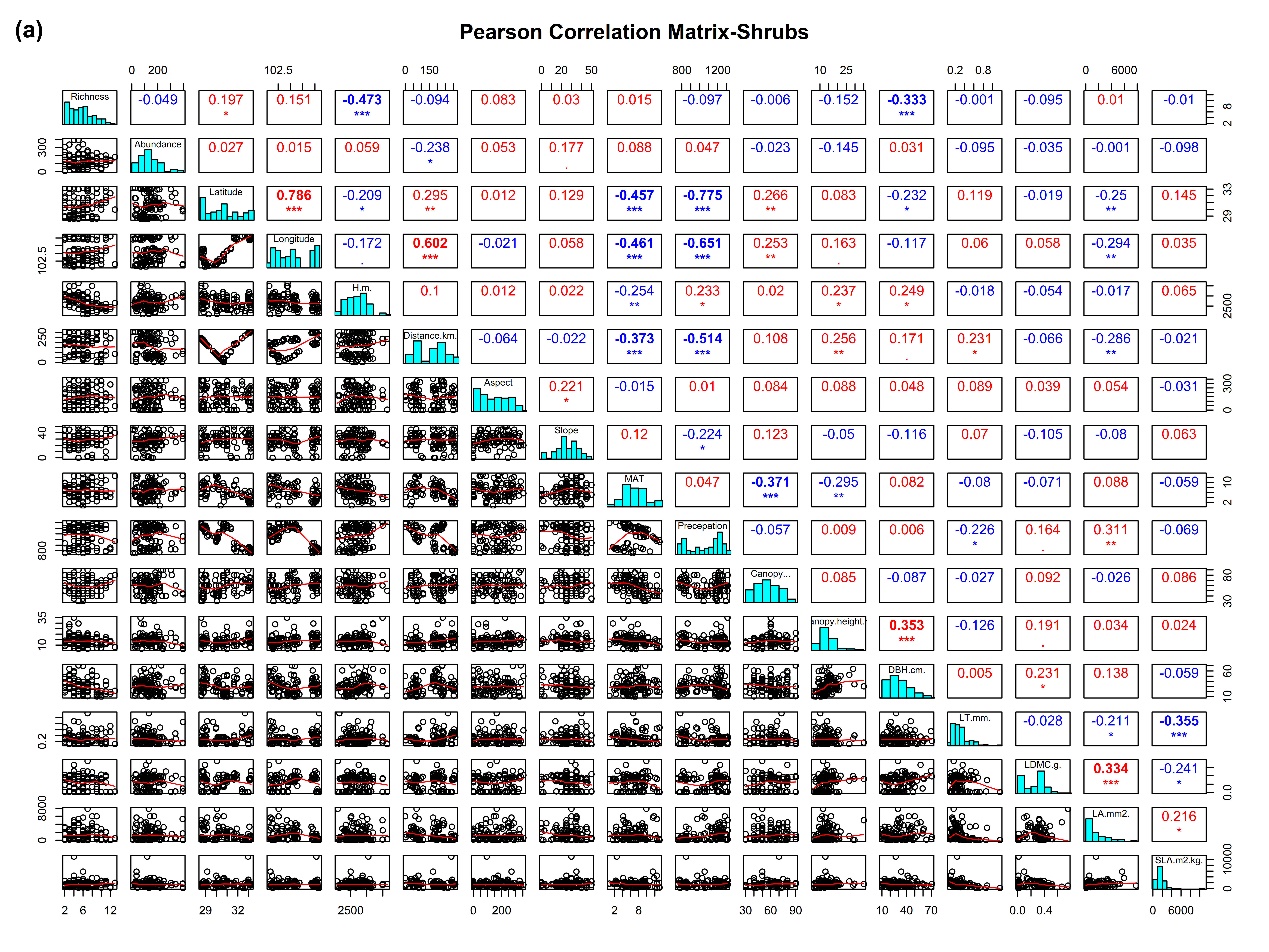


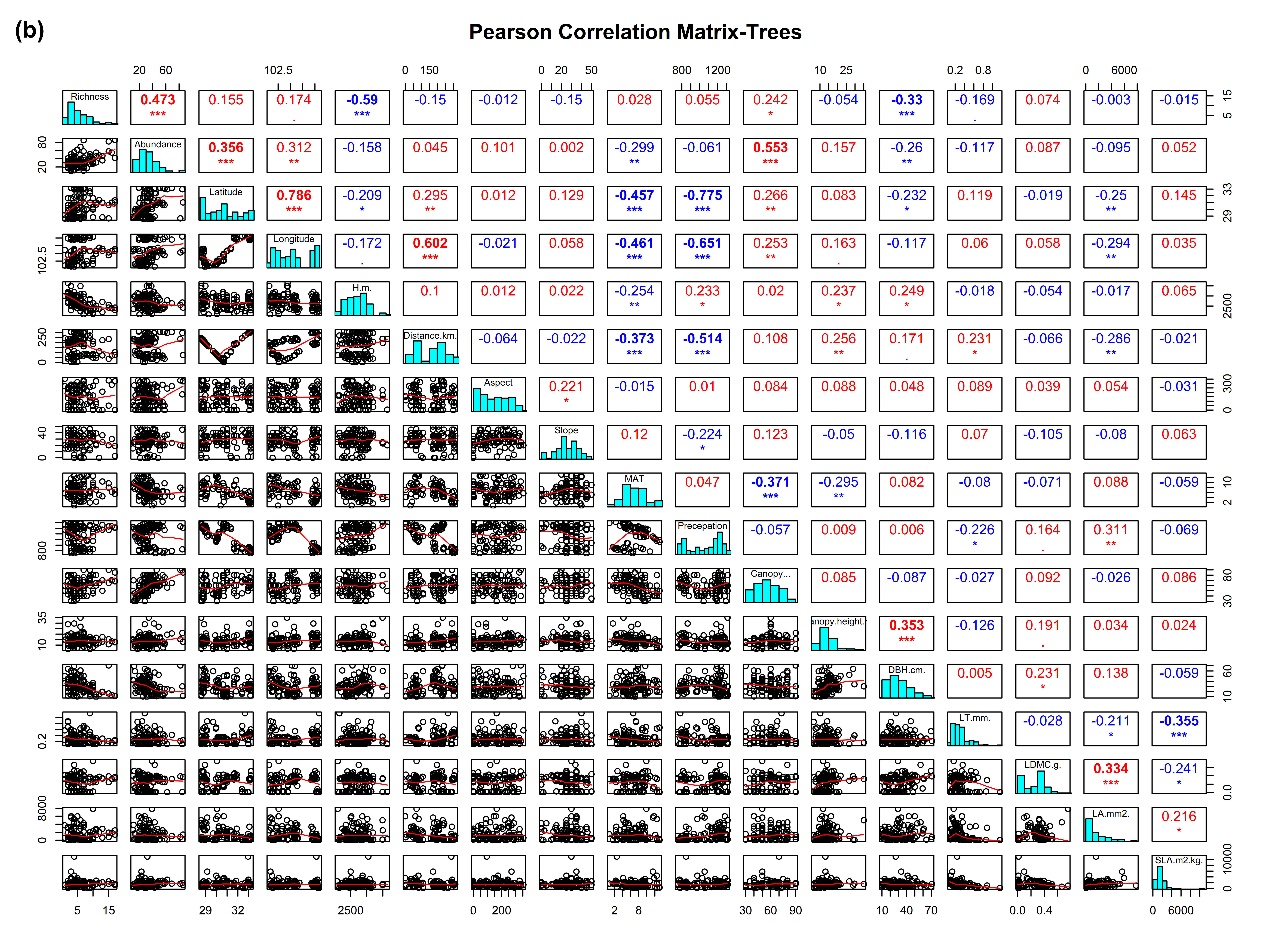


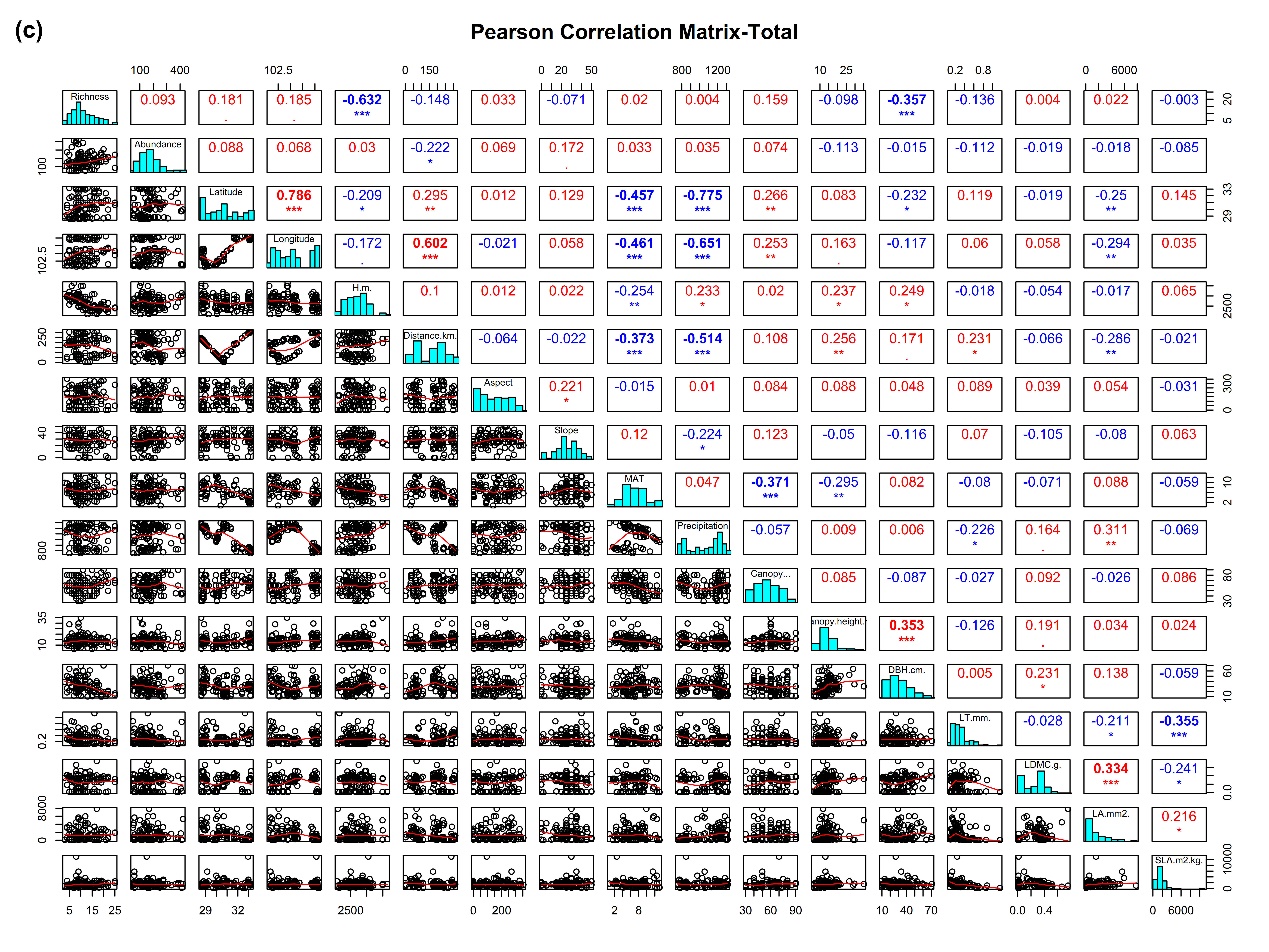


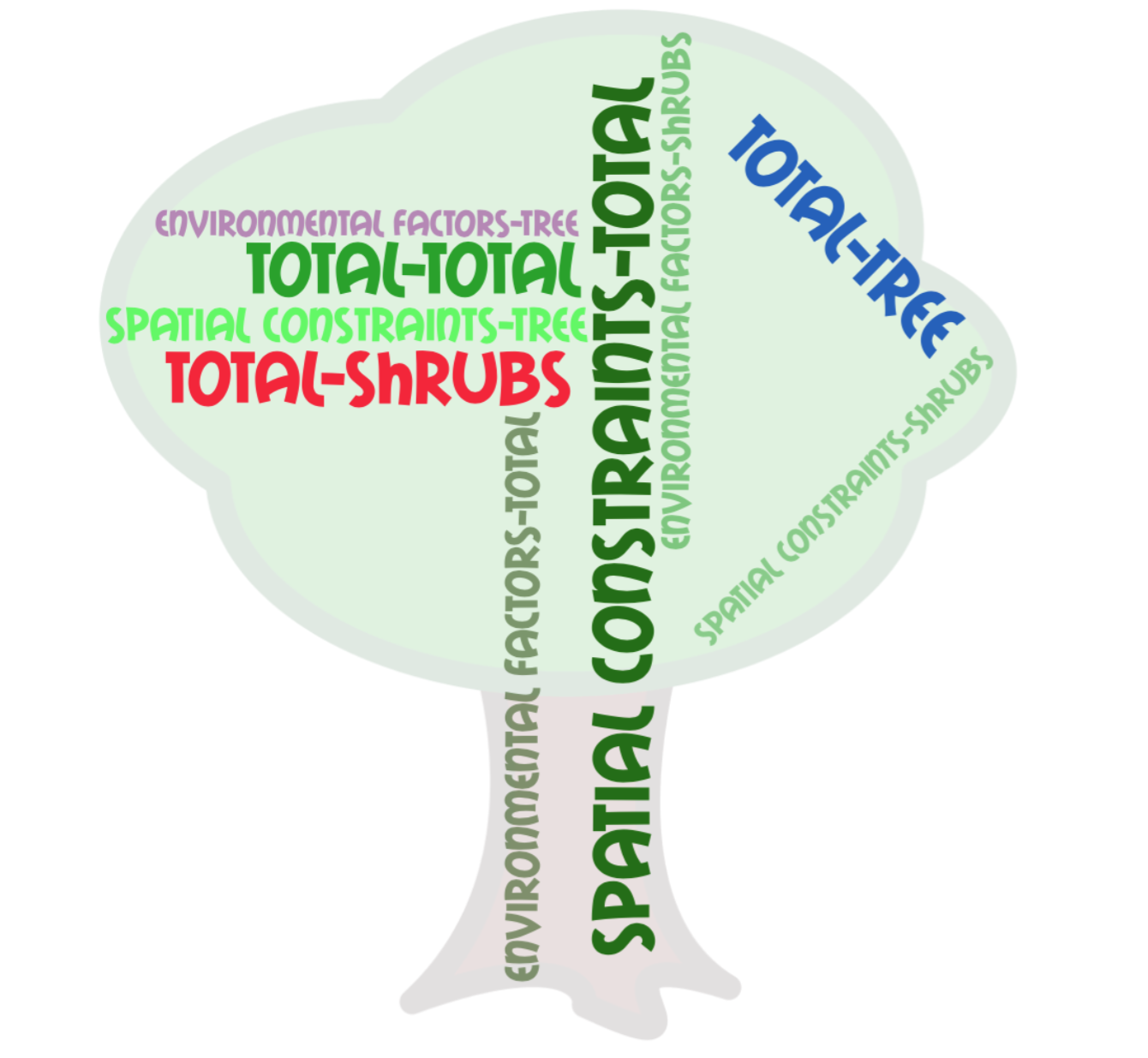


**Fig. S3** Word tree for Mental test results. The font size represents the Pearson correlation coefficient.
