# Supplementary material for: Direct and indirect effects of environmental factors, spatial constraints, and functional traits on shaping the plant diversity of montane forests
Source: Ecol Evol. 2019 Dec 15;10(1):557–68. doi: 10.1002/ece3.5931 (PMC6972828; doi:10.1002/ece3.5931)
Supplement: Supplementary file 2 [file ECE3-10-557-s002.docx]

**Metadata**

Table S2 Environmental, spatial constraints and functional trait variables

| No. | Category | Factor | Abbreviation | Unit |
| --- | --- | --- | --- | --- |
| 1 | Environmental factor | Altitude | H | m |
| 2 |  | Slope |  |  |
| 3 |  | Aspect |  |  |
| 4 |  | Mean annual temperature | MAT | ℃ |
| 5 |  | Precipitation |  | mm |
| 6 | Spatial constraints | Longitude |  |  |
| 7 |  | Latitude |  |  |
| 8 |  | Spatial distance | distance | km |
| 9 | Plant functional traits | Trees canopy coverage | canopy | % |
| 10 |  | Diameter of breast height | DBH | cm |
| 11 |  | Canopy height |  | m |
| 12 |  | Specific leaf area | SLA | cm^2^g^-1^ |
| 13 |  | Leaf area | LA | cm^2^ |
| 14 |  | leaf thickness | LT | mm |
| 15 |  | leaf dry matter content | LDMC | g g^- 1^ |

**Table S3** Model evaluation and selection for the across-forest plot SEMs.

The basic model (Model 1) is our conceptual SEM model shown in Fig. 2a. The nested models are simplified ones, i.e., the previous model minus the path(s) between two variables. For example, Model 6 is equal to Model 5 with the direct pathway from Canopy.height.m.+LT.mm.+DBH.cm.+LA.mm^2^.+ SLA.m^2^.kg. to tree species richness (SR) removed due to their non-significant relationship. The decision to remove a path was mainly based on the *P*-value for the path and the performance of overall model fit.

Model evaluation was determined by the following two criteria: 1) The chi-square test (χ2) (*P* > 0.05 for a satisfactory fit), and 2) The Standardized Root Mean Square Residual (SRMR < 0.05 for a satisfactory fit). The Akaike Information Criterion (AIC) was used to select the best model from models with a satisfactory fit. When a model meets the criteria of the χ2 test and SRMR but contains non-significant paths, we repeat the modeling fit and evaluation by removing these paths. Therefore, the final selected model may not have the minimum AIC value.

**Table S3.1** shrubs

NG.piecewise=list(

SR.model = lm(Richness ~ Abundance+Latitude+Longitude+H.m.+Aspect+Slope + MAT+Precepation+Canopy...+Canopy.height.m.+LT.mm.+DBH.cm.+LDMC.g.+LA.mm2.+SLA.m2.kg., data = NG),

IND.model = lm(Abundance ~Richness+ Latitude+Longitude+H.m.+Aspect+Slope + MAT+Precepation+Canopy...+Canopy.height.m.+LT.mm.+DBH.cm.+LDMC.g.+LA.mm2.+SLA.m2.kg., data = NG),

LT.model=lm(LDMC.g.~H.m.+Aspect+Slope + MAT+Precepation+Canopy.height.m.+LT.mm.+DBH.cm.+Canopy...+LA.mm2.+SLA.m2.kg., data = NG),

CV.model = lm(MAT~Latitude, data = NG),

CV.model1 = lm(Precepation~Latitude, data = NG),

CV.model2 = lm(MAT~Precepation, data = NG)

)

| **Models** | **df** | **χ2** | ***P*** | **SRMR** | **CFI** | **AIC** |
| --- | --- | --- | --- | --- | --- | --- |
| Model 1 * | 21 | 101.65 | 0.00 | 0.064 | 0.73 | 1331.66 |
| Model 2 [Model 1 – (del MAT~alt)] | 22 | 163.77 | 0.00 | 0.095 | 0.53 | 1391.77 |
| Model 3 [Model 2 – (del pre→alt)] | 12 | 120.46 | 0.00 | 0.086 | 0.37 | 1185.42 |
| Model 4[Model 3 – (MAT→preci)] | 1 | 12.86 | 0.00 | 0.017 | 0.85 | 881.84 |
| Model 5[Model 4 – (richness→Longitude+abun+ +Aspect+Slope+functional)] | 12 | 18.47 | 0.10 | 0.022 | 0.92 | 865.45 |
| Model 6 [Model 5 – (abun→Longitude+H.m.+Aspect+Slope+functional)] | 20 | 33.590 | 0.029 | 0.032 | 0.82 | 864.34 |
| **Model 7 [Model 6 – (LDMC→functional)]** | **4** | **4.005** | **0.41** | **0.024** | **1** | **880.45** |

**Table S3.2** Trees

Model 1

NG.piecewise=list(

SR.model = lm(Richness ~ Abundance+Latitude+Longitude+H.m.+Aspect+Slope + MAT+Precepation+Canopy...+Canopy.height.m.+LT.mm.+DBH.cm.+LDMC.g.+LA.mm2.+SLA.m2.kg., data = NG),

IND.model = lm(Abundance ~Richness+ Latitude+Longitude+H.m.+Aspect+Slope + MAT+Precepation+Canopy...+Canopy.height.m.+LT.mm.+DBH.cm.+LDMC.g.+LA.mm2.+SLA.m2.kg., data = NG),

LT.model=lm(Canopy...~H.m.+Aspect+Slope + MAT+Precepation+Canopy.height.m.+LT.mm.+DBH.cm.+LDMC.g.+LA.mm2.+SLA.m2.kg., data = NG),

CV.model = lm(MAT~Latitude, data = NG),

CV.model1 = lm(Precepation~Latitude, data = NG),

CV.model2 = lm(MAT~Precepation, data = NG)

| **Models** | **df** | **χ2** | ***P*** | **SRMR** | **CFI** | **AIC** |
| --- | --- | --- | --- | --- | --- | --- |
| Model 1 | 21 | 90.33 | 0.00 | 0.059 | 0.82 | 1246.72 |
| Model 2 [Model 1 – (del MAT~alt)] | 22 | 152.45 | 0.00 | 0.084 | 0.65 | 1306.84 |
| Model 3 [Model 2 – (del pre~alt)] | 12 | 106.76 | 0.00 | 0.076 | 0.61 | 1100.49 |
| Model 4 [Model 3-(del MAT~pre)] | 1 | 796.91 | 0.32 | 0.005 | 1 | 796.91 |
| Model 5[Model 4 – (abund→longitude+functional)  (richness→longitude+functional)  (canopy→longitude+functional)] | 2 | 5.68 | 0.058 | 0.033 | 0.98 | 775.33 |
| Model 6 [Model 5 –  (richness→abun+aspect)] | 4 | 5.69 | 0.22 | 0.033 | 0.99 | 771.33 |
| Model 7 [Model 6 – (abun→H.m.+Aspect+Slope + MAT)] | 5 | 6.19 | 0.29 | 0.034 | 0.99 | 764.93 |
| **Model 8 [Model 7 – (Canopy→preci)]** | **6** | **6.25** | **0.40** | **0.036** | **0.99** | **762.98** |

**Table S3.3** Total

NG.piecewise=list(

SR.model = lm(Richness ~ Abundance+Latitude+H.m.+Aspect+Slope + MAT+Precipitation+Canopy...+Canopy.height.m.+LT.mm.+DBH.cm.+LDMC.g.+LA.mm2.+SLA.m2.kg., data = NG),

IND.model = lm(Abundance ~Richness+ Latitude+Longitude+H.m.+Aspect+Slope + MAT+Precipitation+Canopy...+Canopy.height.m.+LT.mm.+DBH.cm.+LDMC.g.+LA.mm2.+SLA.m2.kg., data = NG),

LT.model=lm(LDMC.g.~H.m.+Aspect+ Slope + MAT+Precipitation+Canopy.height.m.+LT.mm.+DBH.cm.+Canopy...+LA.mm2.+ SLA.m2.kg., data = NG),

CV.model = lm(MAT~Latitude, data = NG),

CV.model1 = lm(Precipitation~Latitude, data = NG),

CV.model2 = lm(MAT~Precipitation, data = NG)

)

| **Models** | **df** | **χ2** | ***P*** | **SRMR** | **CFI** | **AIC** |
| --- | --- | --- | --- | --- | --- | --- |
| Model 1 * | 22 | 103.32 | 0.00 | 0.067 | 0.77 | 1284.14 |
| Model 2 [Model 1– (del MAT~alt)] | 23 | 165.44 | 0.00 | 0.101 | 0.59 | 1344.26 |
| Model 3 [model2-]– ( del pre~alt) | 13 | 113.74 | 0.00 | 0.088 | 0.52 | 1137.90 |
| Model 4 [Model 3 – (del MAT~pre)] | 2 | 3.62 | 0.16 | 0.007 | 0.99 | 834.33 |
| Model 5 [Model 4 – (richness→abun+Aspect+Slope + MAT+functional)] | 12 | 14.24 | 0.29 | 0.016 | 0.98 | 824.95 |
| Model 6 [Model 5- (abun→Longitude+H.m.+Aspect+functional)] | 17 | 19.99 | 0.28 | 0.027 | 0.97 | 818.73 |
| **Model 7 [Model 6 – (LDMC→H.m.+Aspect+Slope+functional)]** | **6** | **3.94** | **0.69** | **0.022** | **1** | **837.54** |

Notes:

1. ~ Model 2 and Model 1, Model 3 and Model 2, Model 4 and Model 3, Latitude had a significant effect on MAT and precipitation, but in the process of constructing SEM, the paths (MAT/precipitation~ Latitude) were added and SEMs cannot pass the test conditions, so we deleted from this selected model.
2. **→** Delete insignificant impact factor paths, for example, Model 5 and Model 4 both meet the criteria of the χ2 test and SRMR, and Model 6 has a smaller AIC value than the one of Model 7. However, Model 6 contains multiple non-significant paths, which were removed step by step. Thus, Model 7 was finally selected.
3. Bolded models represent the final selected causal models used in the present study.
