# Supplementary material for: Direct and indirect effects of environmental factors, spatial constraints, and functional traits on shaping the plant diversity of montane forests
Source: Ecol Evol. 2019 Dec 15;10(1):557–68. doi: 10.1002/ece3.5931 (PMC6972828; doi:10.1002/ece3.5931)
Supplement: Supplementary file 3 [file ECE3-10-557-s003.docx]

**Table S4** The results of the SEMs including shrub(a), trees(b), total(c)

On the top of our hypothesized relationships among variables (Supplementary Information Fig S2). Latitude had a significant effect on MAT and precipitation, but in the process of constructing SEM, the paths (MAT/precipitation~ Latitude) were added and SEMs cannot pass the test conditions. Therefore, in the SEMs screening process, we removed the paths (MAT/precipitation~ Latitude).

**Metadata**

| **Variable names in SEM outputs** | **Variable names in the SEM conceptual model** | **Meanings** |
| --- | --- | --- |
| Richness | Tree species richness | Species number within plots |
| Abundance | Stem abundance | Stem number within plots |
| LDMC | Leaf dry matter content | Plant community functional traits(Second axis of PCA) |
| Canopy | Trees canopy coverage | Plant community functional traits(First axis of PCA) |
| Lat | Latitude | Geographical factors (First axis of PCA) |
| MAT | Mean annual temperature | Climate (First axis of PCA) |
| Precipitation | Annual precipitation | Climate (First axis of PCA) |
| H | Height above sea level | Topography (Second axis of PCA) |
|  |  |  |
| Regressions (~) |  | Directed path from one variable to the other |
| Variances |  | Unexplained variance of a given variable |
| Estimate |  | Unstandardized path coefficient |
| Std.Err  Std.all |  | SE for unstandardized path coefficient  Standardized path coefficient |

**Table 6.1 Shrubs**

Regressions:

Estimate Std.Err Z-value P(>|z|) Std.lv Std.all

Richness ~

latitude 0.313 0.131 2.388 0.017 0.313 0.313

H -0.483 0.085 -5.656 0.000 -0.483 -0.481

Precipitation 0.285 0.134 2.131 0.033 0.285 0.284

LDMC -0.163 0.084 -1.928 0.054 -0.163 -0.162

Abundance ~

Richness -0.114 0.098 -1.161 0.246 -0.114 -0.114

Lat 0.516 0.208 2.482 0.013 0.516 0.516

MAT 0.306 0.129 2.376 0.017 0.306 0.306

Precipitation 0.422 0.181 2.332 0.020 0.422 0.422

LDMC ~

MAT -0.113 0.099 -1.150 0.250 -0.113 -0.113

Precipitation 0.200 0.098 2.039 0.041 0.200 0.200

H -0.130 0.101 -1.280 0.201 -0.130 -0.130

Variances:

Estimate Std.Err Z-value P(>|z|) Std.lv Std.all

Richness 0.715 0.098 7.280 0.000 0.715 0.718

Abundance 0.925 0.127 7.280 0.000 0.925 0.934

LDMC 0.943 0.130 7.280 0.000 0.943 0.952

**Table 6.2** Trees

Regressions:

Estimate Std.Err Z-value P(>|z|) Std.lv Std.all

Richness ~

Lat 0.522 0.150 3.484 0.000 0.522 0.522

H -0.564 0.076 -7.400 0.000 -0.564 -0.564

Slope -0.131 0.070 -1.883 0.060 -0.131 -0.131

MAT 0.200 0.103 1.941 0.052 0.200 0.201

Precipitation 0.566 0.128 4.426 0.000 0.566 0.566

Canopy 0.237 0.072 3.290 0.001 0.237 0.237

Abundance ~

richness 0.302 0.074 4.065 0.000 0.302 0.305

Lat 0.409 0.118 3.467 0.001 0.409 0.414

Precipitation 0.262 0.116 2.263 0.024 0.261 0.264

Canopy 0.386 0.073 5.258 0.000 0.386 0.390

Canopy ~

MAT -0.391 0.093 -4.212 0.000 -0.391 -0.391

H -0.079 0.093 -0.853 0.394 -0.079 -0.079

Variances:

Estimate Std.Err Z-value P(>|z|) Std.lv Std.all

Richness 0.468 0.064 7.280 0.000 0.468 0.473

Abundance 0.509 0.070 7.280 0.000 0.509 0.526

Canopy 0.848 0.117 7.280 0.000 0.848 0.856**Table 6.3 Total**

Regressions:

Estimate Std.Err Z-value P(>|z|) Std.lv Std.all

Richness ~

latitude 0.441 0.109 4.057 0.000 0.442 0.441

H -0.672 0.071 -9.413 0.000 -0.667 -0.665

Precipitation 0.519 0.111 4.682 0.000 0.519 0.518

LDMC -0.107 0.070 -1.565 0.118 -0.110 -0.109

Abundance ~

Lat 0.566 0.195 2.909 0.004 0.566 0.566

Slope 0.183 0.095 1.930 0.054 0.183 0.183

MAT 0.244 0.124 1.974 0.048 0.244 0.244

Precipitation 0.504 0.174 2.888 0.004 0.504 0.504

LDMC ~

MAT -0.113 0.099 -1.150 0.250 -0.113 -0.113

Precipitation 0.200 0.098 2.039 0.041 0.200 0.200

H -0.130 0.101 -1.280 0.201 -0.130 -0.130

Variances:

Estimate Std.Err Z-value P(>|z|) Std.lv Std.all

Richness 0.493 0.068 7.280 0.000 0.493 0.495

Abundance 0.884 0.122 7.280 0.000 0.886 0.908

LDMC 0.943 0.130 7.280 0.000 0.943 0.952
